# PACT/PRKRA and p53 regulate transcriptional activity of DMRT1

**DOI:** 10.1590/1678-4685-GMB-2019-0017

**Published:** 2020-03-30

**Authors:** Kazuko Fujitani, Asako Otomo, Yuto Nagayama, Taro Tachibana, Rika Kato, Yusuke Kawashima, Yoshio Kodera, Tomoko Kato, Shuji Takada, Kei Tamura, Nobuhiko Takamatsu, Michihiko Ito

**Affiliations:** 1 Kitasato University, Gene Analysis Center, School of Medicine, Sagamihara, Japan.; 2 Tokai University School of Medicine, Department of Molecular Life Sciences, Isehara, Japan.; 3 Osaka City University, Department of Bioengineering, Graduate School of Engineering, Osaka, Japan.; 4 Cell Engineering Corporation, Osaka, Japan.; 5 Kitasato University, Department of Physics, School of Science, Sagamihara, Japan.; 6 National Research Institute for Child Health and Development, Department of Systems BioMedicine, Tokyo, Japan.; 7 Kitasato University, Department of Bioscience, School of Science, Sagamihara, Japan.

**Keywords:** p53, DMRT1, germline stem cell, testis, Xenopus

## Abstract

The transcription factor DMRT1 (doublesex and mab-3 related transcription factor) has two distinct functions, somatic-cell masculinization and germ-cell development in some vertebrate species, including mouse and the African clawed frog *Xenopus laevis*. However, its transcriptional regulation remains unclear. We tried to identify DMRT1-interacting proteins from *X. laevis* testes by immunoprecipitation with an anti-DMRT1 antibody and MS/MS analysis, and selected three proteins, including PACT/PRKRA (Interferon-inducible double-stranded RNA dependent protein kinase activator A) derived from testes. Next, we examined the effects of PACT/PRKRA and/or p53 on the transcriptional activity of DMRT1. In transfected 293T cells, PACT/PRKRA and p53 significantly enhanced and repressed DMRT1-driven luciferase activity, respectively. We also observed that the enhanced activity by PACT/PRKRA was strongly attenuated by p53. Moreover, *in situ* hybridization analysis of *Pact/Prkra* mRNA in tadpole gonads indicated high expression in female and male germline stem cells. Taken together, these findings suggest that PACT/PRKRA and p53 might positively and negatively regulate the activity of DMRT1, respectively, for germline stem cell fate.

## Introduction

DMRT1 (doublesex and mab-3-related transcription factor) belongs to a family of transcription factors characterized by the presence of a DNA-binding domain called DM domain. The *Dmrt1* gene is required for somatic-cell masculinization, which leads to testis formation in various vertebrate species (Yoshimoto *et al.*, 2010; Masuyama *et al.*, 2012; Zhao *et al.*, 2015). The Z-linked *Dmrt1* gene in chicken is involved in male sex determination (Smith *et al.*, 2009). In mice, the loss of *Dmrt1* in adult Sertoli cells reprograms these cells into granulosa cells. Thus, DMRT1 plays an important role in the regulatory networks that maintain masculinization of somatic cells long after the sex determination (Matson *et al.*, 2011). Interestingly, the W-linked *Dm-W* gene in the frog *Xenopus laevis* that we discovered, or the Y-linked *Dmy*/*Dmrt1by* gene in the teleost fish *Oryzias latipes* evolved through whole or partial duplication of *Dmrt1* during diversification of each species for male or female sex determination, respectively (Matsuda *et al.*, 2002; Nanda *et al.*, 2002; Yoshimoto *et al.*, 2008) .

DMRT1 participates not only in somatic cell masculinization, but also germ cell development in some vertebrate species. In mice, DMRT1 negatively controls meiosis in male germ cells, but promotes meiosis in female germ cells (Matson *et al.*, 2010; Krentz *et al.*, 2011). We recently reported that germ cell-specific knockdown of *Dmrt1* caused deficiency of female and male germ stem cells (oogonia and spermatogonia) in *X. laevis*, whereby some genetically female ZW transgenics displayed testicular gonads because of germ cell deficiency (Mawaribuchi *et al.*, 2017a). In addition, our observation of DMRT1 expression in germ cells during *X. laevis* gonadal development suggested that DMRT1 contributes to the maintenance of germline stem cell identity by controlling gene expression (Fujitani *et al.*, 2016).

As for molecular functions of DMRT1 as transcription factor, in mice it represses and enhances *Stra8* transcription in male and female germ cells, respectively (Matson *et al.*, 2010; Krentz *et al.*, 2011). In gonadal somatic cells of mice, DMRT1 could repress transcription of five feminizing genes, *Foxl2, Esr1, Esr2, Wnt4* and *Rspo1* as a repressor, but activates three masculinizing genes *Ptgdr, Sox9*, and *Sox8* as an activator (Matson *et al.*, 2011). To clarify the transcriptional regulation by DMRT1, we tried to isolate DMRT1-associated proteins from *X. laevis* testis extracts by immunoprecipitation with an anti-DMRT1 antibody and mass spectrometry analysis, resulting in the identification of several proteins. Here, we focused on PACT/PRKRA (Interferon-inducible double-stranded RNA dependent protein kinase activator A), because PACT/PRKRA could strongly enhance the transcriptional activity of DMRT1. Because PACT/PRKRA is involved in p53 sumoylation and activation (Bennett *et al.*, 2012), we examined whether the tumor suppressor p53 might be involved in the DMRT1-driven transcriptional regulation. Interestingly, p53 greatly repressed the transcriptional activity of DMRT1, as well as the enhanced activity by PACT/PRKRA in transfected 293T cells.

## Materials and Methods

### Animal care and use

Experimental procedures in the production of monoclonal antibodies were approved by the Institutional Animal Care and Use Committee (permission number: S0036) and performed according to Osaka City University Animal Experimentation Regulations. All of the experiments usinging *X. laevis* were performed under approval by the Institutional Animal Care and Use Committee of Kitasato University (permission number: 1602). *X. laevis* frogs at various developmental stages were purchased from Watanabe Zoushoku (Yachiomachi, Japan) and maintained at 22 °C. Tadpole developmental stages were identified according to the descriptions by Nieuwkoop and Faber (1956).

### Immunogen preparation

A bacterial expression vector pMALc2-DMRT1 (130-336) was constructed by inserting the region encoding residues from 130 to 336 of *X. laevis* DMRT1.L into pMAL-c2 (New England Biolabs). The recombinant protein was produced in *E. coli* Rosetta (DE3) pLysS (Novagen) BL21(DE3), and purified using amylose resin (New England Biolabs), followed by elution with 10 mM maltose, according to the manufacturer’s instructions. The purified protein was dialyzed with phosphate buffered saline (PBS) and used as an immunogen.

### Production of mouse anti-DMRT1 monoclonal antibodies

Mouse monoclonal anti-DMRT1 antibodies were generated based on the mouse medial iliac lymph node method (Sado *et al.*, 2006). Briefly, the purified protein was injected into the tail base with Freund’s complete adjuvant. Three weeks later, cells from the lymph nodes of the immunized mice were fused with mouse myeloma. The resulting hybridoma cells were plated onto 96-well plates and cultured in HAT (hypoxanthine aminopterin thymidine) selection medium. Monoclonal antibodies were purified from the hybridoma supernatants by ion-exchange chromatography.

### Immunoblotting

Samples were run by 10% SDS-PAGE and transferred to FluoroTrans 0.2 μm membrane (PALL). The membrane was blocked with 5% skim milk in PBS, incubated with purified monoclonal antibodies (1:400) or the 1:10,000 diluted anti-DMRT1 rabbit polyclonal antibody (Fujitani *et al.*, 2016) at 4 °C overnight, and then washed with PBST (0.1% Tween-20 in PBS). Next, the samples were incubated with anti-mouse or anti-rabbit IgG-HRP-conjugated secondary antibodies (1 h at RT) and then washed. Signals were detected using ImmunoStar LD substrate (Wako) and C-Digit (LI-COR). Both, the anti-mouse and anti-rabbit HRP-conjugated antibodies, were purchased from SIGMA, and were diluted 1:20,000.

### Immunohistochemistry

Adult testes were dissected and immediately frozen, embedded in FSC 22 Blue compound (Leica), and sliced into 7 μm sections in a cryostat (Leica, CM1850). Sections were fixed in 4% paraformaldehyde (PFA), followed by treatment with 5% bovine serum albumin (BSA), and 0.5% Triton X-100 in PBS. The fixed sections or cells were then incubated overnight with monoclonal antibodies (1:10) or the 1:1000 diluted anti-DMRT1 rabbit polyclonal antibody (Fujitani *et al.*, 2016) at 4 °C. After washing, the sections or cells were incubated with the anti-mouse IgG-Alexa 488 conjugated antibody (1:2,000) and anti-mouse IgG Alexa 592 conjugated antibody (1:2,000), respectively, followed by treatment with 10 μg/mL Hoechst 33258 (Sigma) for nuclei staining. The Alexa-conjugated antibodies were purchased from Invitrogen. Signals were examined by fluorescence microscopy BZ-8000 (Keyence).

### Immunoprecipitation (IP)

Transfected 293T cells or testes dissected from 1-year-old adult *X. laevis* were homogenized in RIPA buffer, followed by sonication. The cell extracts from a 35 mm dish with 1 μg of each anti-DMRT1 monoclonal antibody, or the testicular extracts (10 mg) with 100 μg of the anti-DMRT1 monoclonal antibody 4F6 were mixed with 100 μL of EZveiw Red Protein G Affinity Gel (Sigma), and incubated overnight at 4 ºC. Mouse normal IgG (Santa Cruz Biotechnology; sc-2025) was used as a negative control. The gels were washed twice with RIPA buffer, and the denatured proteins were separated by SDS-PAGE (Perfect NT Gel W, 10–20% acrylamide, 28 wells; DRC Co. Ltd.). Silver staining was performed with the 2D-SILVER STSIN II kit (Cosmo Bio 423413).

### Enzymatic in-gel protein digestion

Gels containing the bands of interest were cut into small pieces, destained in 50% ACN/50 mmol/L NH4HCO3, washed with deionized water, dehydrated in 100% CAN, and dried in an evaporator. The gel pieces were rehydrated in 25 mM Tris-HCl (pH 9.0)/20% ACN containing 50 ng/mL trypsin (sequencing grade; Roche) for 45 min. After unabsorbed solution was removed, the gel pieces were incubated in 50 mM Tris-HCl (pH 9.0) for 20 h at 37 °C. The solution was transferred to a new tube. In addition, the remaining fragments were extracted in 5% formic acid/50% ACN for 20 min at room temperature, and transferred to the tube.

### Protein identification by LC-MS/MS analysis

The digested peptides were desalted and separated by HPLC (the EASY-nLC 1000, Thermo Fisher Scientific) and analyzed by mass spectrometry (Q-Exactive mass spectrometer, Thermo Fisher Scientific). The proteins were identified by using the obtained data and *X. laevis* database.

### cDNA synthesis and RT-qPCR

Isolation of total RNA from *X. laevis* tadpoles and frogs at various stages of development, and cDNA synthesis were performed as described previously (Fujitani *et al.*, 2016). RT-qPCR was carried out using the SYBR Green Realtime PCR Master Mix (ToYoBo, Osaka, Japan). *Pact/Prkra* cDNA was amplified using the following primer pair: 5’-CAGCTGCTGCATGAATTTG-3’ (forward) and 5’-CTCTCCTAAGCTAGTTATGTCACC-3’ (reverse).

### cDNA cloning and plasmid construction

*phb2*, *yb-1*, and *Pact/Prkra* cDNAs were amplified from *X. laevis* adult testis cDNA by PCR using PrimeSTAR polymerase (TaKaRa) with the following primer sets: *phb2* (5’-GCTCAGAATTTAAAGGATTTTGC-3’, 5’-TCACT TCTTTCCTTGTTTGAAAAC-3’), *yb-1* (5’-AGCAGC GAGGTTGAAACAC-3’, 5’-TTACTCAGCCCCGCCCT G-3’) and *PACT/PRKRAPact/Prkra* (5’-TCCCAGGAG AGGTTTCCAG-3’, 5’-TCACTTTTTAATACACATG ATTTTTA-3’), respectively. PCR products were cloned into a vertebrate expression vector pcDNA3-S-Tag (Ito *et al.*, 1999). Effector plasmids used for luciferase reporter assay pcDNA3-FLAG-p53 were cloned into the vertebrate expression vector pcDNA3-FLAG (Ito *et al.*, 1999) by PCR using Prime STAR polymerase (TaKaRa) with the following primer pair (5’-GAACCTTCCTCTGAGAC-3’, 5’-TCATTCCGAGTCGGGCTGTTC-3’).

### Luciferase reporter assay

Twenty four hours before transfection, 293T cells were plated at 510^4^ cells per well in a 48-well plate. The cells were transfected with luciferase reporter plasmid p4xDMRT1-luc (Yoshimoto *et al.*, 2010), effector plasmids, and *Renilla* luciferase vector pRL-SV40 (Promega) by PEI MAX vector. After 24 hours, luciferase activities were measured in a Luminocounter 700 (Niti-ON). Firefly luciferase activity was normalized by *Renilla* luciferase activity using the dual luciferase assay system (Promega).

### Whole mount *in situ* hybridization

Whole mount *in situ* hybridization for *X. laevis Pact/Prkra* mRNA was performed as described previously (Wada *et al.*, 2017), using DIG-labeled sense or anti-sense probes from nucleotides 1-930 in GenBank number NM_001086031.1.

### Statistical analysis

Two-group, or multiple group comparisons were performed by Student’s *t*-test or one-way ANOVA followed by Tukey HSD test, respectively. Significance for all tests was set at *p* < 0.05.

## Results

### Mouse monoclonal antibody 4F6 reacts specifically to *X. laevis* DMRT1

**Figure 1:**
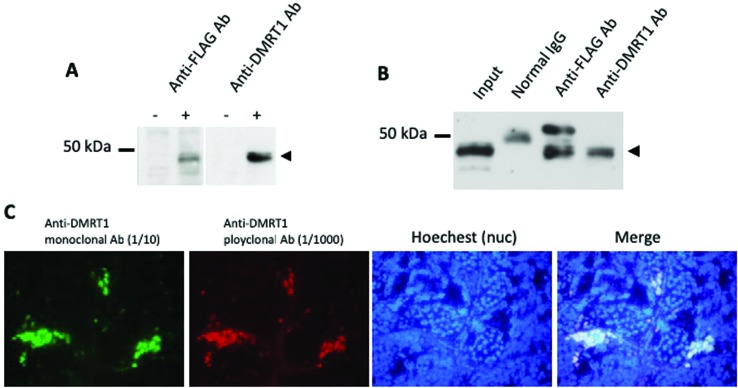
Immunoreaction of an anti-DMRT1 monoclonal antibody 4F6 against *X. laevis* DMRT1. (A) Immunoblot analysis using the anti-FLAG (M5) or anti-DMRT1 (4F6) monoclonal antibodies. pcDNA3-FLAG or pcDNA3-FLAG-DMRT1 was transiently transfected into 293T cells. Extracts of 293T cells were examined by immunoblotting with each antibody followed by a HRP conjugated anti-mouse IgG antibody. DMRT1 was detected as a single band at the same size by both antibodies (arrowhead). (B) Immunoprecipitation (IP) analysis with the anti-FLAG (M5) or anti-DMRT1 (4F6) monoclonal antibodies. pcDNA3-FLAG-DMRT1 was transiently transfected into 293T cells. The cell lysate was mixed with each antibody and pulled down with protein A/G agarose. IP extracts were examined by immunoblotting using the anti-DMRT1 polyclonal antibody followed by a HRP conjugated anti-rabbit IgG antibody. (C) Immunohistochemical analysis using the anti-DMRT1 monoclonal antibody 4F6 (1/10) and anti-DMRT1 polyclonal antibodies (1/1000) . Frozen sections of adult testis were stained with Hoechst 33258 for nuclei and reacted with both the antibodies, followed by Alexa 594-conjugated anti-mouse (red) and Alexa 488 anti-rabbit-IgG (green) antibodies. Both signals showed the same staining patterns.

To identify DMRT1-associating proteins in *X. laevis* testes by proteome analysis, we produced mouse monoclonal antibodies against the truncated C-terminal protein of *X. laevis* DMRT1 from 130 to 336 aa, which contains a specific region among DM domain family proteins. We examined the specificity of 20 monoclonal antibodies to DMRT1 by immunoblotting, immunoprecipitation (IP) and immunohistochemistry (IHC), and from this screen we selected the antibody 4F6. The results using 4F6 are shown in Figure 1. 4F6 reacted specifically to overexpressed FLAG-tagged DMRT1 in 293T cells on immunoblot analysis (Figure 1A). Immunoprecipitates using 4F6 from the extract of 293T cells overexpressing FLAG-tagged DMRT1 showed a specific reaction with the anti-FLAG antibody (Figure 1B). In addition, the IHC analysis with 4F6 or the anti-DMRT1 polyclonal antibody (Fujitani *et al.*, 2016) on sections of adult testis revealed that both antibodies reacted to the exact same cells, that is, spermatogonia and Sertoli cells (Figure 1C).

### PACT/PRKRA has the potential to enhance transcriptional activity of DMRT1

**Figure 2:**
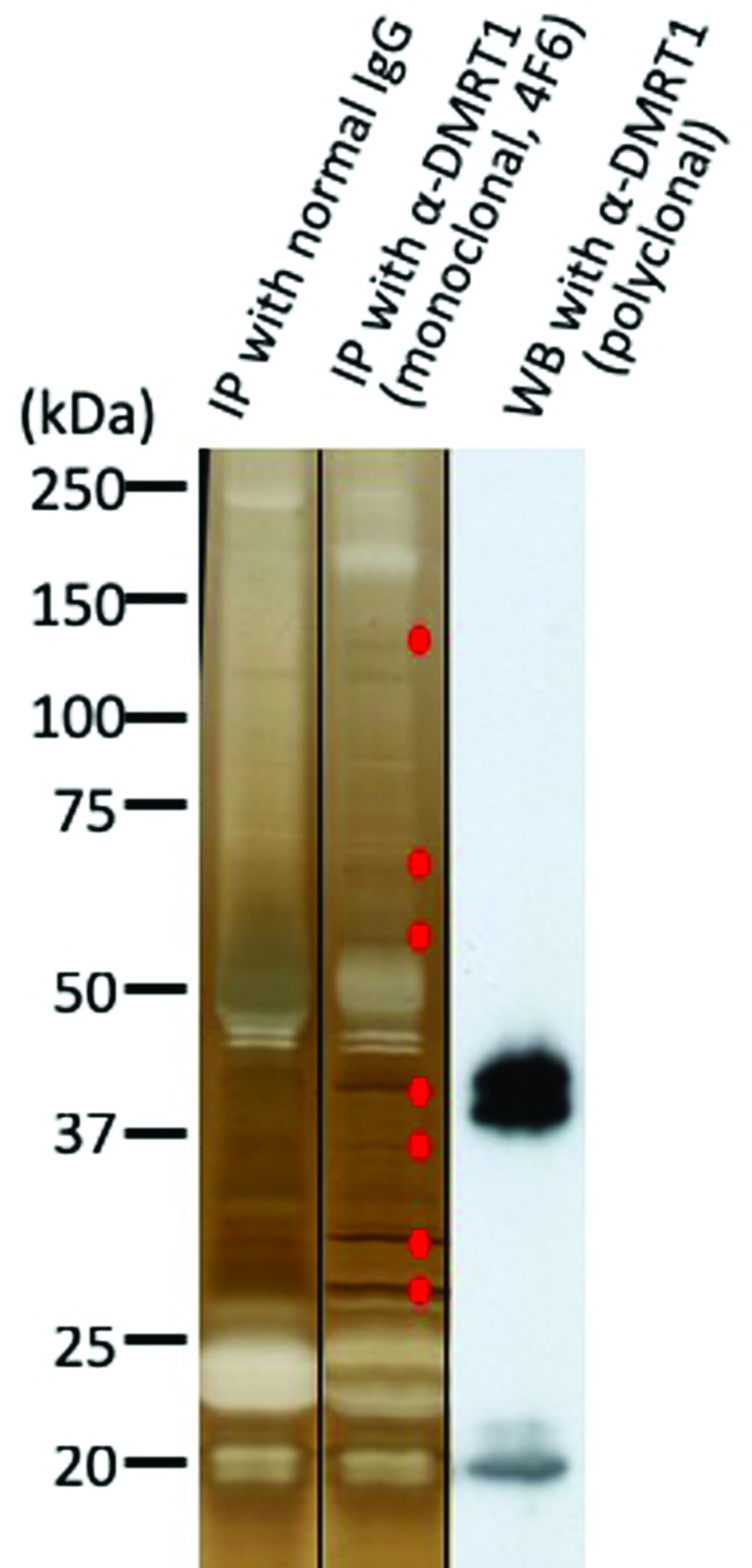
Silver staining of immunoprecipitates using the anti-DMRT1 monoclonal antibody 4F6 from *X. laevis* adult testes. Testis extracts were mixed with normal mouse IgG or the anti-DMRT1 antibody 4F6, and pulled down with protein A/G agarose. The immunoprecipitates were examined by silver staining (the right and middle lanes) or immunoblotted with an anti-DMRT1 polyclonal antibody (right lane). Seven 4F6-specific bands were excised, and examined by LC-MS.

Immunoprecipitates obtained by the anti-DMRT1 monoclonal antibody 4F6 and normal mouse IgG as a negative control from *X. laevis* adult testes were separated by SDS-PAGE, followed by silver staining. We compared the staining patterns between the two IP samples, and observed seven bands specific to 4F6 (Figure 2). Each band derived from 4F6 and its corresponding region derived from normal IgG were excised from the gels and analyzed by mass spectrometry. We identified 332 proteins from the 4F6 IP sample. From these, 124 proteins were selected as 4F6-specific proteins, because the remaining 208 proteins were also found in the sample using normal IgG. We then focused on three proteins, Prohibitin 2 (PHB2), Y-box binding protein-1 (YB-1), and PACT/PRKRA, which are all known to function in nuclei.

**Figure 3:**
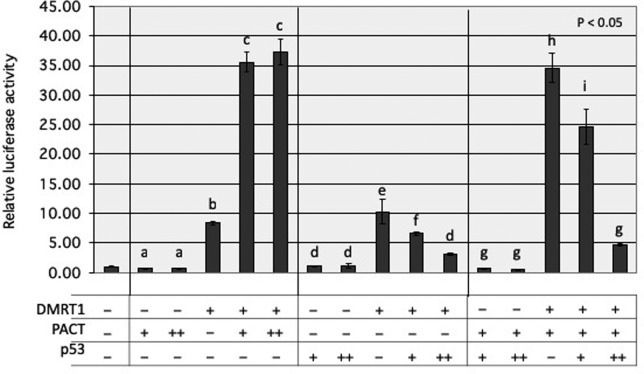
Effects of PACT/PRKRA and/or p53 on transcriptional activity by DMRT1 using luciferase reporter assay. 150 ng of DMRT1-driven firefly luciferase reporter plasmid (p4xDMRT1-luc), 3.3 ng of DMRT1 expression plasmid (pcDNA3-FLAG-DMRT1), and 10 ng Renilla luciferase vector (pRL-TK-luc) as transfection internal control in the presence or absence of PACT/PRKRA and/or p53 expression plasmids (pcDNA3-FLAG-PACT/PRKRA and/or -p53) were transiently co-transfected into 293T cells, using 1.2 μg PEI MAX. Total amount of DNA was kept at 250 ng per each transfection with pcDNA3-FLAG empty vector. 24 hours after transfection, cell lysates were used to measure luciferase activity. Relative activity is shown as the fold increase compared with the value obtained with 250 ng of pcDNA3-FLAG empty vector. The symbols -, +, and ++ indicate 0, 3.3, and 20 ng, respectively. Values are expressed as mean ± SE, n = 3. The letters above the bars indicate the results of Tukey HSD test following one-way ANOVA (*p* < 0.05).

To clarify how PHB2, YB-1, and PACT/PRKRA are involved in DMRT1 function, we investigated the effect of each protein on transcriptional regulation by DMRT1 using the luciferase reporter assay. Expression plasmids for each protein and DMRT1, as well as a DMRT1-driven luciferase reporter plasmid carrying four repeats of the DMRT1-binding sequence 5’-TTGATACATTGTTGC-3’ (Yoshimoto *et al.*, 2010) were co-transfected into 293T cells (Figure S1). Exogenous expression of PHB2 had a small effect and YB-1 a slightly stronger effect on luciferase activities driven by DMRT1. In contrast, PACT/PRKRA greatly enhanced the DMRT1-driven activity in a dose-dependent manner (Figure 3 and Figure S1).

We also examined whether each protein could directly interact with DMRT1 in cultured cells. After co-expression of DMRT1 and S-tagged PHB2, YB-1, and PACT/PRKRA in 293T cells, the cell extracts were mixed with S-protein agarose. Then, the pull-down samples as well as the cell extracts were examined by western blot analysis (data not shown). No signals for DMRT1 bound to PHB2, YB-1, or PACT/PRKRA could be detected, indicating the possibility of indirect interactions with DMRT1.

### p53 has the potential to repress transcriptional activity of DMRT1

PACT/PRKRA was characterized as a negative regulator of p53 (Li *et al.*, 2007). Thus, we investigated the effects of p53 on transcriptional activity of DMRT1 in the presence and absence of exogenous PACT/PRKRA using the luciferase reporter assay in co-transfected 293T cells (Figure 3). The DMRT1-driven luciferase activity enhanced by PACT/PRKRA was found strongly and dose-dependently down-regulated by p53 expression. Interestingly, even in the assay without exogenous PACT/PRKRA, the DMRT1-driven activity was also significantly repressed by p53 expression in a dose-dependent manner.

### *Pact/Prkra* mRNA is expressed in germline stem cells of the tadpole gonads

**Figure 4:**
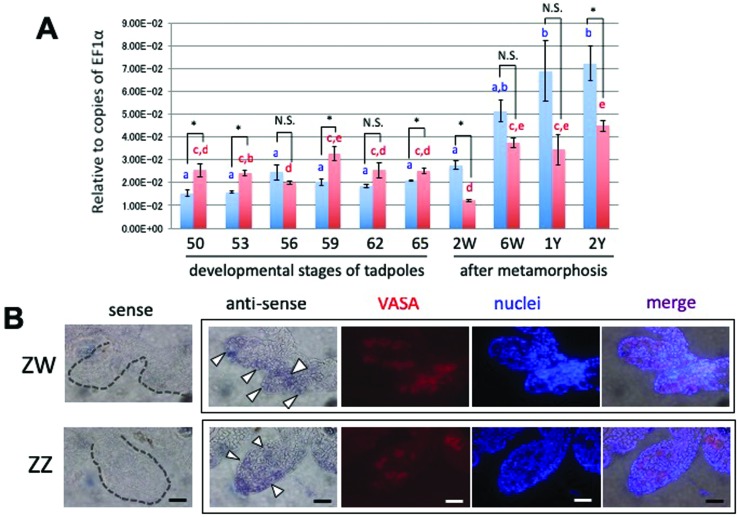
Expression of *PACT/PRKRA* mRNA in developing ZW and ZZ gonads. (A) Quantitative RT-PCR analysis of *Pact/Prkra* mRNA during gonadal development of ZW (red) and ZZ (blue) tadpoles and adults in *X. laevis*. cDNAs were synthesized using total RNAs from ZW and ZZ gonads at various stages of tadpoles after sex determination, and at 6 weeks, 1 year, and 2 years of frogs after metamorphosis, and then amplified by PCR using specific primer pairs as described in Table S1. W and Y show weeks and year(s), respectively. *EF1*α was used for normalization. RT-qPCR data represent the mean (n=3) and SD. Values are expressed as mean ± SE, n = 3. Differences among stages were evaluated by one-way ANOVA followed by the Tukey HSD test (*p* < 0.05). Mean values not sharing the same letters are significantly different from each other. Sexual differences between ZZ and ZW gonads at each stage were evaluated or by Student’s *t*-test (*p < 0.05). N.S., not significant. (B) Distribution of *Pact/Prkra* mRNAs on transverse sections of ZW and ZZ tadpole gonads at stage 56. Whole-mount *in situ* hybridization of the gonads with the attached mesonephros was performed with the *Pact/Prkra* sense or anti-sense RNA probe, followed by 7-μm cryostat sectioning. The sections were treated with an anti-VASA monoclonal antibody to identify germ cells (red) and Hoechst 33258 for nuclei (blue). Note that the nuclei in germ cells were faintly stained by Hoechst 33258. Arrowheads indicate *Pact/Prkra*-expressing germ cells. Scale bars, 20 μm.

Because *Pact/Prkra* can contribute to upregulation of DMRT1 function, we next investigated the expression profile of *Pact/Prkra* mRNA in developing gonads in *X. laevis*. We first performed an RT-qPCR analysis of *Pact/Prkra* transcripts during gonadal development (Figure 4A). The *Pact/Prkra* transcripts showed no or only few significant differences between ZW and ZZ gonads from stage 50, just after sex determination, to stage 65, when metamorphosis is almost completely finished. In addition, the transcripts of ZW or ZZ gonads exhibited uniform expression during tadpole development. In contrast, the amount of the *Pact/Prkra* mRNA gradually increased in adult testes from 6 weeks after metamorphosis to 1-2 years, which might be related to a prosperous spermatogenesis.

Next, to clarify the topological distribution of the *Pact/Prkra* mRNA, we used DIG-labeled *Pact/Prkra* RNA sense and anti-sense probes to perform a whole mount *in situ* hybridization on stage 56 ZW and ZZ tadpole gonads, which started displaying sexual differences in morphology. To topologically identify *Pact/Prkra*-expressing cells, we analyzed sections that were counterstained with an anti-VASA antibody and the nuclear stain Hoechst 33258 (Figure 4B). Germline stem cells are characterized as not only expressing of VASA, but also faint staining of their nuclei. The anti-sense probe seemed to hybridize to *Pact/Prkra* mRNA in both somatic and germ cells of the ZW and ZZ gonads, while almost no signals were detected with the sense probe. Strong signals were observed in some germline stem cells of both sexes.

## Discussion

For comprehending gonadal development, including sex determination and differentiation, in the African clawed frog *X. laevis* carrying a ZZ/ZW-type sex-determining system, we previously identified *Dmrt1* and its W-linked paralog *Dm-W*, and characterized the former as a gene for testis formation and germ-cell development and the latter as a female sex-determining gene (Yoshimoto *et al.*, 2006, 2008, 2010; Yoshimoto and Ito, 2011; Fujitani *et al.*, 2016; Mawaribuchi *et al.*, 2017a). We also reported on the molecular evolution of *Dmrt1* family genes (Mawaribuchi *et al.*, 2012, 2017a b). However, it remained unknown how DMRT1 activates or represses transcription of its target genes in gonadal somatic cells and germ cells as a transcription factor. In this study, to understand the transcriptional regulation by DMRT1, we tried to identify such functions of DMRT1 in *X. laevis* by analyzing immunoprecipitates with an anti-DMRT1 monoclonal antibody from extracts of adult testes. From the more than one hundred identified proteins we selected three, PHB2, YB-1, and PACT/PRKRA for further analysis.

Unexpectedly, a protein-protein binding assay in co-transfected 293T cells indicated that these proteins apparently do not directly interact with DMRT1 (data not shown). Rather, the result suggested that each protein might be indirectly associated with DMRT1 through other DMRT1-binding proteins. PHB2 is an intercellular communicator between nucleus and mitochondria, and suppresses transcription of target genes in nuclei (Bavelloni *et al.*, 2015). In contrast, the transcription factor YB-1 is involved in transcriptional regulation by interacting with other transcription factors, including p53 (Okamoto *et al.*, 2000). Strikingly, exogenous expression of PHB2 or YB-1 induced only few or no changes in our DMRT1-driven luciferase reporter assay (Figure S1), indicating that transcription driven by PHB2 or YB-1 could be by indirect interaction with DMRT1.

In the DMRT1-driven luciferase reporter assay using transfected 293T cells, only PACT/PRKRA enhanced the luciferase activity significantly (Figure 3 and Figure S1). PACT/PRKR is also known as RAX, P2P-R, and RBBP6. Importantly, p53 significantly repressed the enhanced activity by PACT/PRKRA (Figure 3), indicating a p53-PACT/PRKRA and DMRT1-PACT/PRKRA interaction. We also found that p53 could moderately attenuate the DMRT1-driven activity in the absence of exogenous expression of PACT/PRKRA. Because we could not observe a direct interaction between DMRT1 and p53 in co-transfected 293T cells (data not shown), p53 might also indirectly participate in the PACT/PRKRA-independent transcription by DMRT1.

The next two questions we asked were: In what types of cells does PACT/PRKRA enhance transcriptional activity by DMRT1 or p53 repress its enhanced activity? And what does the regulation by PACT/PRKRA and/or p53 mean? *In situ* hybridization analysis showed that PACT/PRKRA was highly expressed in female and male germline stem cells (oogonia and spematogonia) in tadpole gonads of *X. laevis* (Figure 4B). This observation coincided with the expression pattern during gonadal development in ZW and ZZ tadpoles and adults (Figure 4A). We recently reported that, in *X. laevis* germline stem cells, DMRT1 and a phosphorylated form of the histone variant H2AX (γH2AX) could contribute to the maintenance of their stem cell identity and participate in genome protection against double strand breaks, respectively (Fujitani *et al.*, 2016). p53 has been described as “the guardian of the genome”, because it plays important roles in cell cycle regulation, DNA repair, and apoptosis, leading to genome stability by preventing mutations or eliminating DNA-damaged, mutated cells. For the next generation, p53 functions to guarantee germ cell quality (Takashima *et al.*, 2013; Gebel *et al.*, 2017), as in mouse spermatogonial stem cells, *Dmrt1* depletion causes apoptosis, but both *Dmrt1* and *p53* depletion induces pluripotency, suggesting that p53 and DMRT1 might play contrary and/or related roles in spermatogonial stem cells. In other words, the balance between DMRT1 and p53 might maintain germline stem cell identity. Taken together, these findings suggest that PACT/PRKRA might enhance DMRT1 function for germline stem cell identity, but p53 negatively controls DMRT1 function, leading to apoptosis in damaged, mutated germline stem cells. In addition, YB-1, one of the three DMRT1-interacting proteins identified in this study, was found to directly bind with p53 (Okamoto *et al.*, 2000), as described above. It is possible that germline stem cell identity regulated by DMRT1 and p53 might be mediated not only through PACT/PRKRA, but also YB-1.

PACT/PRKRA has been characterized as a dsRNA binding protein (Redfern *et al.*, 2013), a RISC (RNA-induced silencing complex) member required for subsequent siRNA-mediated post-transcriptional gene silencing (Patel and Sen, 1998), and an activator of protein kinase R (PKR), also known as interferon-induced, dsRNA-activated protein kinase (Huang *et al.*, 2002). We presently have no indication on whether and how dsRNA/RISC may be involved in the PACT/PRKRA-DMRT1 interaction. Interestingly, PKR is associated with p53 (Cuddihy *et al.*, 1999), and PACT/PRKRA-PKR signaling in response to stress-inhibited p53 turnover lead to G1 cell cycle arrest (Bennett *et al.*, 2012). It will be interesting to clarify whether the two signaling modules, PACT/PRKRA-PKR-p53 and DMRT1-PACT/PRKRA-p53, have mutual relation in germline stem cells.

## Supplementary material

The following online material is available for this article:

Figure S1 - Effects of PHB2, YB1, and PACT/PRKRA on transcriptional activity by DMRT1 using luciferase reporter assay.

Table S1 - Sequences of oligonucleotide primers used in PCR reaction for cloning.
