# Supplementary material for: PACT/PRKRA and p53 regulate transcriptional activity of DMRT1
Source: Genet Mol Biol. 2020 Mar 30;43(2):e20190017. doi: 10.1590/1678-4685-GMB-2019-0017 (PMC7198010; doi:10.1590/1678-4685-GMB-2019-0017)
Supplement: Supplementary file 1 [file 1415-4757-GMB-43-2-e20190017-s1.pdf]

*Genetics and Molecular Biology* 43, 2 (2020)

# Supplementary Material to "PACT/PRKRA and p53 regulate transcriptional activity of DMRT1"

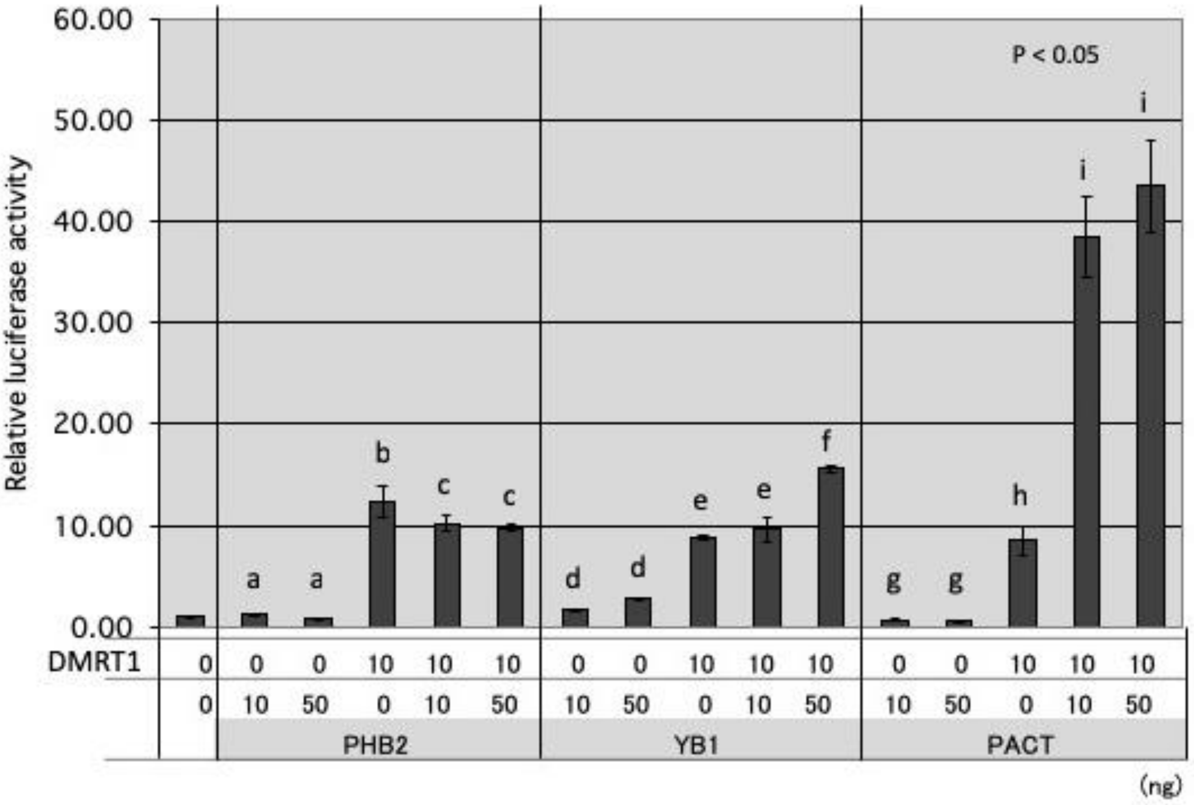


**Figure S1** - Effects of PHB2, YB1, and PACT/PRKRA on transcriptional activity by DMRT1 using luciferase reporter assay. DMRT1-driven firefly luciferase reporter assay was performed by almost the same method as described in the legend of Figure 3. Ten nanograms of DMRT1 expression plasmid (pcDNA3-FLAG-DMRT1) in the presence (10 or 50 ng) or absence of PHB2, YB1, or PACT/PRKRA expression plasmids were transiently co-transfected into 293T cells.
