# Supplementary material for: PACT/PRKRA and p53 regulate transcriptional activity of DMRT1
Source: Genet Mol Biol. 2020 Mar 30;43(2):e20190017. doi: 10.1590/1678-4685-GMB-2019-0017 (PMC7198010; doi:10.1590/1678-4685-GMB-2019-0017)
Supplement: Supplementary file 2 [file 1415-4757-GMB-43-2-e20190017-s2.pdf]

Supplementary Material to “PACT/PRKRA and p53 regulate transcriptional activity of DMRT1”

Table S1 - Sequences of oligonucleotide primers used in PCR reaction for cloning.

construct	forward primer	reverse primer
pMALc2-DMRT1 (130-336)	5'-CCACTTCAGGATCCACAGCCA-3'	5'-aaactcgagCTATTCTCCATTTTCAGCAG-3'
pcDNA3-FLAG-DMRT1	5'-aaaggatccATGCAAAACAATGAGGAACC-3'	5'-aaactcgagCTATTCTCCATTTTCAGCAG-3'
pcDNA3-S-PRKRA	5'-aaaaagcttTCCCAGGAGAGGTTTCCAG-3'	5'-aaactcgagTCACTTTTTAATACACATGATTTTTA-3'
pcDNA3-S-PHB2	5'-aaagaattcgGCTCAGAATTTAAAGGATTTTGC-3'	5'-aaactcgagTCACTTCTTTCCTTGTTTGAAAAC-3'
pcDNA3-EGFP-PHB2	5'-aaagaattcATGGCTCGAATTTAAAGGA-3'	5'-aaactcgagCACTTCTTTCCTTGTTTGAAAAC-3'
pcDNA3-EGFP-CACTTCTTT	5'-aaagaattcATGAAAGAGTCCGTATTTACAGTGG-3'	5'-aaactcgagCACTTCTTTCCTTGTTTGAAAAC-3'
pcDNA3-S-FLOT2	5'-aaacaattcGGGGAACTGCCATACAGTGG-3'	5'-aaactcgagTTACGCCATGATGCCTGTG-3'
pcDNA3-S-PKR	5'-aaaggatccATGGATGATCTGAATGTCAAAG-3'	5'-aaaactcgagTTAATGGGTTTTTGAGTCCAAG-3'
pcDNA3-FLAG-p53	5'-ATGGAACCTTCCTCTGAGAC-3'	5'-TCATTCCGAGTCGGGCTGTTC-3'
